# Supplementary material for: Impaired skeletal muscle mitochondrial pyruvate uptake rewires glucose metabolism to drive whole-body leanness
Source: eLife. 2019 Jul 18;8:e45873. doi: 10.7554/eLife.45873 (PMC6684275; doi:10.7554/eLife.45873)
Supplement: Figure 3—source data 1. — (n = 8, littermates, age = 15 weeks, two way ANOVA). Data are presented as mean ± SEM (**p<0.01, ***p<0.001). [file elife-45873-fig3-data1.docx]

| **Source data 1** | | | | | | | |
| --- | --- | --- | --- | --- | --- | --- | --- |
|  |  |  |  |  |  |  |  |
|  |  | **12 hr fast** | | **Refed** | | |  |
|  |  | WT | SkmKO | WT | SkmKO | |  |
| **Serum** | **Insulin** | 0.44 ± 0.02 | 0.52 ± 0.05 | 0.68 ± 0.03 | 0.81 ± 0.11 | | ng/mL |
|  | **Triglycerides** | 67.73 ± 6.48 | 63.63 ± 1.55 | 57.70 ± 4.49 | 46.25 ± 1.93 | | mmol/L |
|  | **NEFAs** | 0.89 ± 0.06 | 0.94 ± 0.06 | 0.41 ± 0.04 | 0.34 ± 0.04 | | mEq/L |
|  | **Cholesterol** | 106.01 ± 3.32 | 97.86 ± 3.95 | 96.57 ± 1.71 | 92.45 ± 2.82 | | mg/dL |
|  | **Ketones** | 1396.04 ± 74.46 | 1084.19 ± 123.88** | 266.05 ± 34.04 | 168.52 ± 17.88 | | µmol/L |
|  | **Lactate** | 1.61 ± 0.11 | 1.85 ± 0.14 | 3.43 ± 0.22 | 4.25 ± 0.49 | | mmol/L |
|  | **Glucose** | 114.12 ± 4.99 | 111.12 ± 9.51 | 390.25 ± 20.90 | 297.62 ± 17.66*** | | mg/dL |
|  |  |  |  |  |  | |  |
|  |  | Data are mean ± SEM; **p<0.01, ***p<0.001 | | | |  |  |
