# Supplementary material for: Impaired skeletal muscle mitochondrial pyruvate uptake rewires glucose metabolism to drive whole-body leanness
Source: eLife. 2019 Jul 18;8:e45873. doi: 10.7554/eLife.45873 (PMC6684275; doi:10.7554/eLife.45873)
Supplement: Figure 6—source data 1. — (n = 8, littermates, age 58 and 71 weeks, two way ANOVA). Data are presented as mean ± SEM (*p<0.05, **p<0.01, ***p<0.001). [file elife-45873-fig6-data1.docx]

| **Source data 4** | | | | | | | | | | | |
| --- | --- | --- | --- | --- | --- | --- | --- | --- | --- | --- | --- |
|  |  |  |  |  |  |  |  |  |  |  |  |
| **Serum parameters post 48 weeks on HFD** | | | | | | | | | |  | |
|  |  | | **12 hr fast** | | | **Refed** | | | |  | |
|  |  | | WT | SkmKO | | WT | | SkmKO | |  | |
| **Serum** | **Insulin** | | 3.2 ± 0.42 | 3.07 ± 0.42 | | 6.16 ± 0.75 | | 3.44 ± 0.3*** | | ng/mL | |
|  | **Triglycerides** | | 39.51 ± 1.48 | 35.6 ± 2.07 | | 44.63 ± 2.03 | | 35.02 ± 2.69* | | mmol/L | |
|  | **NEFAs** | | 0.59 ± 0.05 | 0.51 ± 0.02 | | 0.37 ± 0.05 | | 0.43 ± 0.06 | | mEq/L | |
|  | **Cholesterol** | | 168.88 ± 10.59 | 179.09 ± 12.83 | | 170.94 ± 14.88 | | 162.34 ± 10.58 | | mg/dL | |
|  | **Ketones** | | 771.08 ± 54.92 | 612.31 ± 70.37 | | 161.29 ± 40.38 | | 183.1 ± 31.13 | | µmol/L | |
|  | **Lactate** | | 1.05 ± 0.12 | 1.46 ± 0.17 | | 1.82 ± 0.16 | | 4.44 ± 0.81*** | | mmol/L | |
|  | **Glucose** | | 144.0 ± 18.3 | 148.33 ± 12.20 | | 205.25 ± 12.92 | | 213.75 ± 18.34 | | mg/dL | |
|  |  | |  |  | |  | |  | |  | |
|  |  | | Data are mean ± SEM; *p<0.05, ***p<0.001 | | | | |  | |  | |
| **Serum parameters post 14 weeks on NFD** | | | | | | | | | | |  |
|  |  | **12 hr fast** | | | | | **Refed** | | | |  |
|  |  | WT | | | SkmKO | | WT | | SkmKO | |  |
| **Serum** | **Insulin** | 1.07 ± 0.24 | | | 0.65 ± 0.10 | | 1.49 ± 0.39 | | 0.90 ± 0.14 | | ng/mL |
|  | **Triglycerides** | 59.65 ± 6.16 | | | 35.69 ± 2.92*** | | 50.64 ± 4.42 | | 29.45 ± 2.97** | | mmol/L |
|  | **NEFAs** | 0.63 ± 0.06 | | | 0.54 ± 0.06 | | 0.31 ± 0.04 | | 0.23 ± 0.03 | | mEq/L |
|  | **Cholesterol** | 129.45 ± 12.82 | | | 016.23 ± 21.74 | | 115.64 ± 7.38 | | 107.72 ± 20.12 | | mg/dL |
|  | **Ketones** | 1017.94 ± 58.54 | | | 920.92 ± 38.67 | | 307.63 ± 63.48 | | 171.90 ± 50.11 | | µmol/L |
|  | **Lactate** | 1.07 ± 0.07 | | | 1.43 ± 0.08 | | 3.78 ± 0.48 | | 3.96 ± 0.33 | | mmol/L |
|  | **Glucose** | 104.75 ± 4.34 | | | 104.5 ± 7.88 | | 167.75 ± 20.78 | | 174.3 ± 8.86 | | mg/dL |
|  |  |  | | |  | |  | |  | |  |
|  |  | Data are mean ± SEM; **p<0.01, ***p<0.001 | | | | | | |  | |  |
